# Prions in Milk from Ewes Incubating Natural Scrapie

**DOI:** 10.1371/journal.ppat.1000238

**Published:** 2008-12-12

**Authors:** Caroline Lacroux, Stéphanie Simon, Sylvie L. Benestad, Séverine Maillet, Jacinthe Mathey, Séverine Lugan, Fabien Corbière, Hervé Cassard, Pierrette Costes, Dominique Bergonier, Jean-Louis Weisbecker, Torffin Moldal, Hugh Simmons, Frederic Lantier, Cécile Feraudet-Tarisse, Nathalie Morel, François Schelcher, Jacques Grassi, Olivier Andréoletti

**Affiliations:** 1 UMR INRA ENVT 1225, Interactions Hôte Agent Pathogène, Ecole Nationale Vétérinaire de Toulouse, Toulouse, France; 2 CEA, Service de Pharmacologie et d'Immunoanalyse, IBiTec-S, DSV, CEA/Saclay, Gif sur Yvette, France; 3 National Veterinary Institute, Sentrum, Oslo, Norway; 4 INRA Domaine de Langlade, Pompertuzat, France; 5 VLA Weybridge, New Haw, Addlestone, Surrey, United Kingdom; 6 INRA IASP, Centre INRA de Tours, Nouzilly, France; Istituto Superiore di Sanità, Italy

## Abstract

Since prion infectivity had never been reported in milk, dairy products originating from transmissible spongiform encephalopathy (TSE)-affected ruminant flocks currently enter unrestricted into the animal and human food chain. However, a recently published study brought the first evidence of the presence of prions in mammary secretions from scrapie-affected ewes. Here we report the detection of consistent levels of infectivity in colostrum and milk from sheep incubating natural scrapie, several months prior to clinical onset. Additionally, abnormal PrP was detected, by immunohistochemistry and PET blot, in lacteal ducts and mammary acini. This PrP^Sc^ accumulation was detected only in ewes harbouring mammary ectopic lymphoid follicles that developed consequent to Maedi lentivirus infection. However, bioassay revealed that prion infectivity was present in milk and colostrum, not only from ewes with such lympho-proliferative chronic mastitis, but also from those displaying lesion-free mammary glands. In milk and colostrum, infectivity could be recovered in the cellular, cream, and casein-whey fractions. In our samples, using a Tg 338 mouse model, the highest per ml infectious titre measured was found to be equivalent to that contained in 6 µg of a posterior brain stem from a terminally scrapie-affected ewe. These findings indicate that both colostrum and milk from small ruminants incubating TSE could contribute to the animal TSE transmission process, either directly or through the presence of milk-derived material in animal feedstuffs. It also raises some concern with regard to the risk to humans of TSE exposure associated with milk products from ovine and other TSE-susceptible dairy species.

## Author Summary

A decade ago, a new variant form of Creutzfeldt-Jakob disease was identified. The emergence of this prion disease in humans was the consequence of the zoonotic transmission of bovine spongiform encephalopathy through dietary exposure. Since then, the control of human exposure to prions has become a priority, and a policy based on the exclusion of known infectious materials from the food chain has been implemented. Because all investigations carried out failed to reveal evidence of infectivity in milk from affected ruminants, this product has continuously been considered as safe. In this study, we demonstrate the presence of prions in colostrum and milk from sheep incubating natural scrapie and displaying apparently healthy mammary glands. This finding indicates that milk from small ruminants could contribute to the transmission of prion disease between animals. It also raises some concern with regard to the risk to humans associated with milk products from ovine and other dairy species.

## Introduction

Transmissible spongiform encephalopathies (TSE), or prion disease, are fatal neurodegenerative disorders occurring in sheep (scrapie), cattle (bovine spongiform encephalopathy - BSE), or humans (Creutzfeldt-Jakob disease - CJD). The key event in TSE is the conversion of a normal cellular protein (PrP^c^) into an abnormal isoform (PrP^Sc^) which accumulates in tissues in infected individuals. According to the prion concept, abnormal PrP is the causative agent of TSEs [1] and PrP^Sc^ is currently considered to be the only TSE biochemical marker. Whereas its detection generally correlates with the presence of infectivity [2],[3], infectivity has been reported in the absence of detectable PrP^Sc^ [4].

A decade ago, a new variant form of CJD was identified. The emergence of this TSE form in humans was the consequence of the zoonotic transmission of BSE through dietary exposure to contaminated animal products [5],[6]. Since then, the control of human exposure to TSE agents has become a priority, and a sanitary policy has been implemented based on both the eradication of TSE in food producing animals and the exclusion of known infectious materials from the food chain.

Because investigations carried out as early as the 1960's failed to reveal evidence of TSE agents in milk from affected ruminants, this product has continuously been considered as safe [7],[8]. However, more recently, disease associated prion protein (PrP^Sc^) accumulation was reported in mammary glands from three scrapie-affected ewes. Deposits were associated with those mammary ectopic lymphoid follicles that develop in response to retroviral infection (Maedi) [9],[10]. Nevertheless, in the absence of definitive evidence of the presence of prion in milk, dairy products originating from TSE affected ruminant flocks continue to enter the animal and human food chain.

In the past few months, however, evidence for the transmission of scrapie to lambs, via colostrum/milk, has been reported [11]. These data raise new concerns about the potential infectious character of milk from TSE affected small ruminants flocks.

## Results

In this study, we first investigated material collected between 2003 and 2006 from a sheep flock with a high incidence of natural scrapie (Langlade Flock) [12] and in which Maedi lentivirus has been endemic for more than 10 years. Sheep from this flock were investigated for the presence of PrP^Sc^ in (i) mammary glands, (ii) lymphoreticular system (LRS) and (iii) central nervous system (CNS) (Table 1). The sheep carryied various PRP polymorphisms at codons 136, 154 and 171 that are associated either with high susceptibility (A_136_R_154_Q_171_/VRQ- VRQ/VRQ– ARQ/ARQ) or resistance (homozygote and heterozygote ARR) to TSEs [12]. The majority of susceptible genotype animals were clinically suspect of scrapie at the time of culling.

**Table 1:** PrP^Sc^ in central nervous system, lympho-reticular system, mammary gland and milk duct lumen of natural scrapie exposed ewes bearing various genotypes at codons 136, 154 and 171 of the PRP gene.

Genotype	number	PrP^Sc^ in obex	PrP^Sc^ in tonsil, prescapular lymph node, spleen and mammary lymph node	PrP^Sc^ in mammary gland	Ectopic mammary lymphoid follicles	PrP^Sc^ in lacteal ducts
**VRQ/VRQ**	n = 110	pos	pos	pos n = 45	n = 45	n = 27
				neg n = 65	n = 0	n = 0
**ARQ/VRQ**	n = 11	pos	pos	pos n = 2	n = 2	n = 1
				neg n = 9	n = 0	n = 0
**ARQ/ARQ**	n = 13	pos	pos n = 6	pos n = 3	n = 3	n = 1
				neg n = 3	n = 0	n = 0
			neg n = 7	neg n = 7	n = 3	n = 0
**ARR/VRQ**	n = 9	pos	neg	neg	n = 3	n = 0
**ARR/ARR**	n = 32	neg	neg	neg	n = 16	n = 0

All investigated animals (n = 175) were PCR positive for Maedi lentivirus. Abnormal PrP (PrP^Sc^) detection was carried out using Western Blotting (SHa31 anti-PrP antibody), ELISA (TeSeE Sheep and Goat, Bio-Rad) and immunohistochemistry (8G8 anti-PrP antibody). The majority of susceptible genotype sheep (ARQ/VRQ- VRQ/VRQ and ARQ/ARQ) were clinical suspect for scrapie at culling.

From within a population of 175 ewes, PCR positive for Maedi virus, 72 displayed lesions of lympho-proliferative chronic mastitis. Of these, PrP^Sc^ positive mammary glands were observed only in sheep showing ectopic mammary lymphoid follicles (n = 50) and then only in those PrP genotypes harbouring LRS PrP^Sc^.

As previously described, in some (n = 7/13) susceptible genotypes (ARQ/ARQ) [13],[14] and in all (n = 9) investigated heterozygote ARR scrapie incubating ewes, no PrP^S^ was detected in LRS, despite its typical accumulation in CNS [15],[16],[17],[18] (Table 1). In those animals, no PrP^Sc^ was detected in mammary glands, even when ectopic lymphoid follicles were present. This observation supports the contention that ewes bearing the ARR allele, even when incubating scrapie, are less likely to shed TSE agent in their milk.

**Figure 1:**
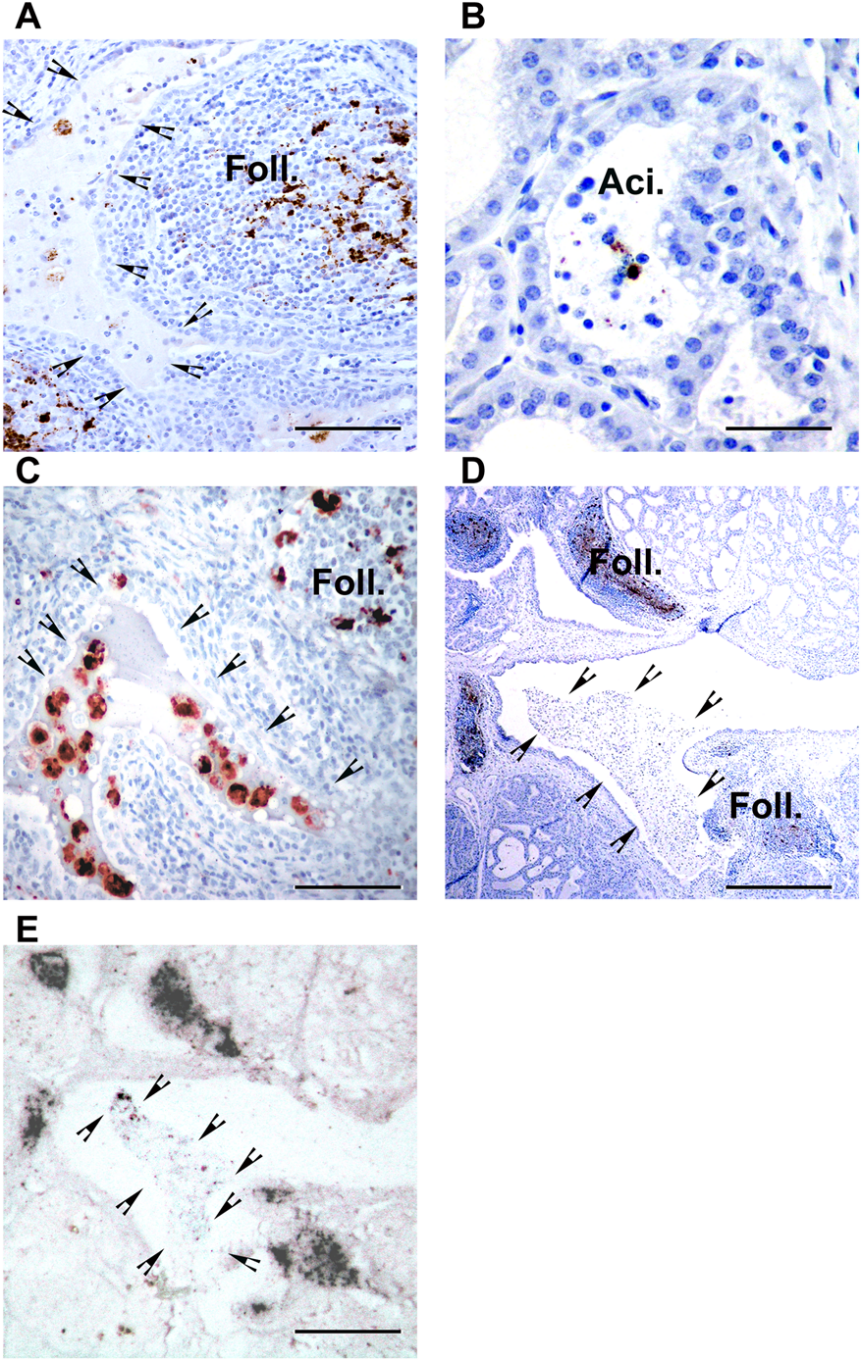
PrP^Sc^ detection in mammary gland from scrapie-incubating sheep. (A) PrP^Sc^ immunolabelling (8G8 monoclonal antibody- DAB brown deposit – bar: 80 µm) in mammary gland from a ewe incubating scrapie (preclinical phase – 15 months old – ARQ/ARQ genotype) and harbouring lympho-proliferative mastitis with ectopic lymphoid follicles (Foll.). In the milk ducts lumen (arrow heads), several PrP^Sc^ positive cells are identifiable. (B) In mammary gland acini (Aci.), positive PrP^Sc^ staining can be observed; either associated with cells or distributed as free granules. (C) Double labelling for PrP^Sc^ (R521 polyclonal serum – black deposits) and CD68 (KiM6 clone – red deposits) indicates that intracellular PrP^Sc^ in milk ducts and acini lumen is associated with phagocytic cells. (D) PrP^Sc^ immunolabelling (8G8 anti-PrP antibody – DAB brown deposit- bar: 200 µm) and (E) PET blot (SHa31 antibody – NBT/BCIP black deposits – bar: 200 µm) of two successive mammary gland sections confirmed that material in milk ducts is proteinase K resistant (arrow heads indicate lining).

In PrP^Sc^ positive mammary glands abnormal PrP accumulation occurred mainly in ectopic lymphoid follicles (n = 50). However, in approximately half of these cases (n = 29), PrP^Sc^ positive cells or free granules were observed in milk ducts and in the lumen of acini. Such pictures were observed not only in clinically affected scrapie ewes but also in animals showing no scrapie clinical signs at the time of culling (Figure 1A and 1B).

Double labelling indicated that these PrP^Sc^ positive cells were also positive with CD68, a marker of phagocyte cells which could encompass both macrophages and dendritic cell subsets [16],[19] (Figure 1C). A PET blot confirmed that the PrP^Sc^ positive elements, observed by immunohistochemistry in milk ducts and acini lumen, were proteinase K resistant (Figure 1D and 1E). The presence of PrP^Sc^ positive material in acini lumen and lacteal ducts was observed in VRQ/VRQ, ARQ/VRQ and ARQ/ARQ animals, indicating that PrP^Sc^ shedding in milk is not restricted to a particular genotype (Table 1).

In a second phase of the study, colostrum and milk were collected from negative control ewes (Arthur Rickwood TSE free flock, UK; n = 5) and scrapie incubating ewes (n = 13) belonging to the Langlade flock. All animals were either of the ARQ/VRQ or VRQ/VRQ PrP genotype. Sampling was carried out during the ewes' first lactation period (13–15 months old), at which age, in the Langlade natural scrapie infection model, VRQ/VRQ sheep show a consistent PrP^Sc^ deposition in both LRS and CNS. However, whereas in ARQ/VRQ sheep of the same age, PrP^Sc^ deposition can be detected in all LRS structures, CNS involvement remains marginal (Table 2). Ewes included in this prospective part of the study were followed up until clear scrapie clinical signs were noted (respectively at 22 and 34 months old in the VRQ/VRQ and ARQ/VRQ ewes). They were then investigated *post-mortem* for the presence of ectopic lymphoid follicles in mammary tissue and for PrP^Sc^ distribution elsewhere.

**Table 2:** PrP^Sc^ distribution in the organism of scrapie incubating animals.

Organ/Age	4 Months				7 Months				10 Months				13 Months				20 Months				32 Months	
	VRQ/VRQ		ARQ/VRQ		VRQ/VRQ		ARQ/VRQ		VRQ/VRQ		ARQ/VRQ		VRQ/VRQ		ARQ/VRQ		VRQ/VRQ		ARQ/VRQ		ARQ/VRQ	
**Obex**	−		−		−		−		4	+/++	−		4	+++	1(a)	+	4	++++	4	++	4	**++++**
**Tonsil**	4	++++	−		4	++++	3(a-b-c)	+/++	4	++++	4	+++	4	++++	4	++++	4	**++++**	4	++++	4	**++++**
**Spleen**	4	++	−		4	+++	2(a-b)	+	4	++++	3(a-b-c)	+	4	++++	4	+++	4	**++++**	4	++++	4	**++++**
**Duodenal PP**	4	++++	1(a)	+	4	++++	4	++	4	++++	4	++	4	**++++**	4	++++	4	++++	4	+++	4	++++
**Jejunum PP**	4	++++	1(c)	+	4	++++	4	++	4	++++	4	**+++**	4	**++++**	4	++++	4	++++	4	++++	4	++++
**Ileum PP**	4	++++	3(a-b-c)	++	4	++++	4	+++	4	**++++**	4	**++++**	4	**++++**	4	++++	4	++++	4	++++	4	++++
**Caecum PP**	4	+++	−		4	++++	3(a-b-c)	+	4	++++	4	**++**	4	**++++**	4	++++	4	++++	4	++++	4	++++
**NLM-jejunal**	4	+++	−		4	++++	4	++	4	++++	4	**+++**	4	**++++**	4	++++	4	++++	4	++++	4	++++
**Ileal MLN**	4	+++	2(a-b)	+	4	++++	4	+++	4	++++	4	**++**	4	**++++**	4	++++	4	++++	4	++++	4	++++
**Médiastinal LN**	4	+++	−		4	+++	3(a-b-c)	+	4	++++	4	++	4	++++	4	++++	4	**++++**	4	++++	4	**++++**
**Prescapular LN**	4	++	−		4	+++	3(a-b-c)	+	4	++++	4	+	4	++++	4	++++	4	**++++**	4	++++	4	**++++**
**Retro hepatic LN**	4	++	−		4	++++	2(a-b)	+	4	++++	4	+	4	++++	4	++++	4	**++++**	4	++++	4	**++++**
**AbomasumENS**	−		−		−		−		1(c)	++	−		1(d)	**++**	−		4	+++	1(a)	++	3(a-c-d)	+++
**DuodenumENS**	−		−		1(a)	0/+	−		4	+++	1(c)	0/+	4	**+++**	4	+/++	4	+++	4	++	4	+++
**Ileum ENS**	−		−		4	++	−		4	**+++**	4	**+**	4	**+++**	4	++	4	+++	4	+++	4	+++
**Caecum ENS**	−		−		3(a-b-d)	+	−		4	++	2(a-c)	**+**	4	**+++**	3(a-b-c)	+	4	+++	4	+++	4	+++
**Colon ENS**	−		−		1(b)	+	−		4	++	−		4	**+++**	2(a-c)	+	4	+++	4	+++	4	+++

Groups of 4 ARQ/VRQ and 4 VRQ/VRQ sheep (named a-b-c-d) were killed at different time of the incubation period. Clinical signs occurred at 20 months in VRQ/VRQ animals and at 32 months in ARQ/VRQ. A systematic PrP^Sc^ detection was realized using immunohistochemistry (8G8 antibody) in a large panel of the collected sheep tissues. PrP^Sc^ accumulation level was scored according to a semi-quantitative scale: (−) no PrP^Sc^, (+) minimal PrP^Sc^ deposits, (++), (+++) moderate PrP^Sc^ deposits and (++++) strong PrP^Sc^ deposits.

**LN**: Lymph Node; **MLN**: Mesenteric Lymph Node; **PP**: Peyer's Patches; **ENS**: Enteric Nervous System.

**Figure 2:**
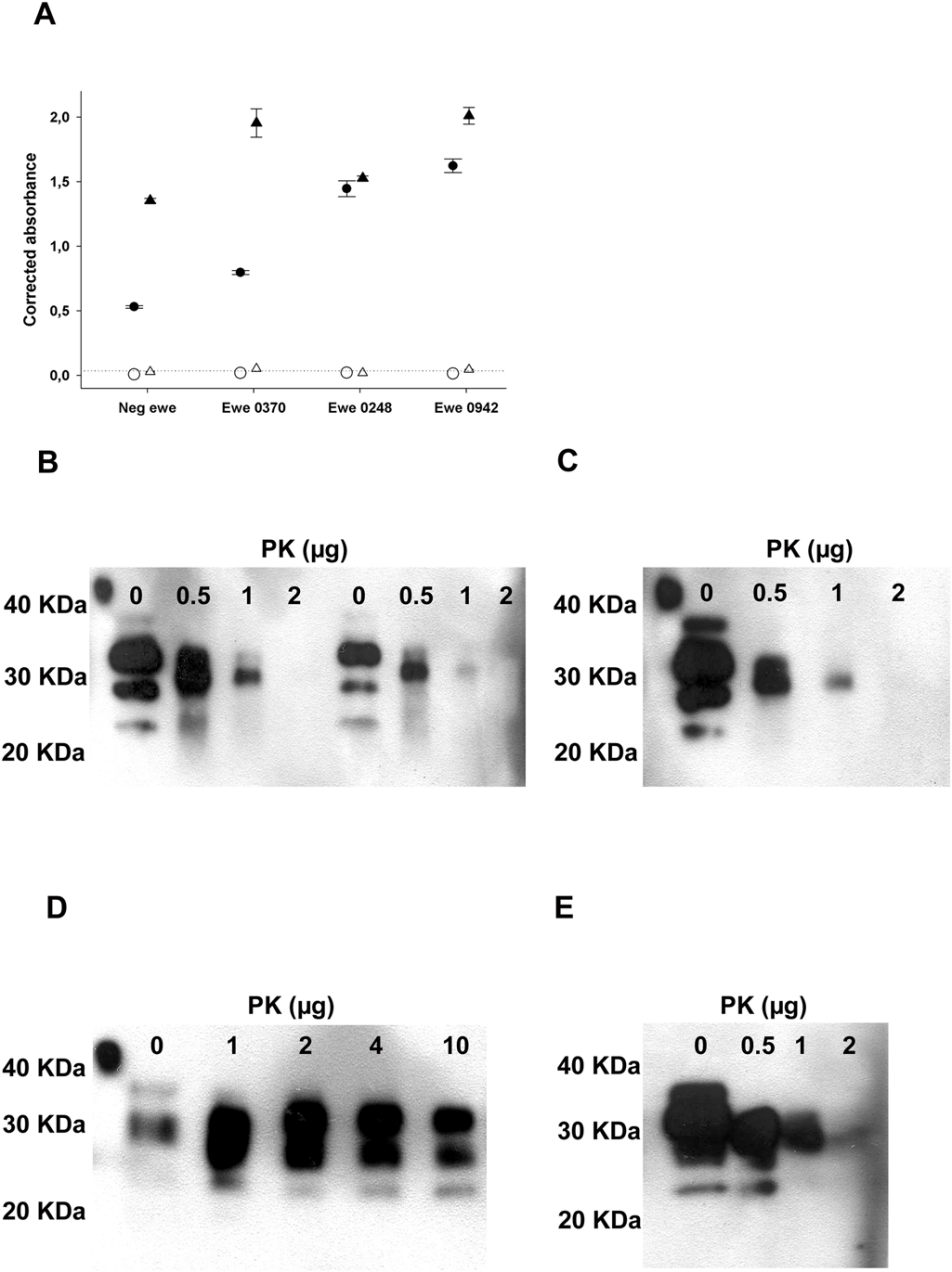
Immunoprecipitation of PrP in milk and colostrum. (A) PrP in milk (▵) and colostrum (○), from a negative control animal and three scrapie incubating sheep (casein whey protein extract following NP40/DOC – 10 min at 37°C treatment). PRP levels were measured before (black symbols) and after (white symbols) immunoprecipitation with antibodies (SHa31, SAF-34, and βS-36). The dosage was performed using a two-site sandwich immunoassay (capture antibody 11C6, tracer antibody Bar-224). The positive threshold of the test (0.040 absorbance units) is symbolised by the dotted line. (B–E) PrP contained in different fractions was immunoprecipitated with Sha31/SAF-34/BS36 immunobeads. After washings, PK in PBS (0 to 10 µg in 50 µL) was added to the beads for 10 min at 37°C. Samples were denatured in laemmli's buffer (25 µL), without β-mercaptoethanol, for 5 min at 100°C. Supernatants were then analysed by western blot. (B) 1.4 mL of casein whey, prepared from colostrum (left four lanes) or milk (right four lanes of the gel), from a scrapie incubating ewe (0942 see Table 4), (C) 1.4 mL of casein whey prepared from a TSE free control milk, (D) 100 µl of scrapie positive 2% brain homogenate or (E) 100 µl of scrapie negative 2% brain homogenate.

Samples were fractionated into cream, casein-whey and cellular pellets. All fractions from all animals were subjected to an immuno-precipitation process, using magnetic beads coated with a mixture of three anti PrP antibodies (SAF32, βS36, SHa31) [20],[21]. This method allowed the concentration of all detectable PrP forms contained in 300 µL of cream and 600 µL of whey into a 20 µL volume, providing an important concentration factor before bioassay (15 times concentration for cream and 30 for whey) (Figure 2). No PK resistant PrP was detected in any sample by Western Blot of bead eluates (Figure 2).

PrP containing beads were then intra-cerebrally (20 µL per mouse) inoculated into transgenic mice over-expressing ovine VRQ PrP (Tg338) [22]. With the Langlade scrapie isolate, according to the end point titration of brain material, incubation periods can reach up to 850 days in Tg338 mice (Table 3). Consequently, for the majority of colostrum and milk samples, bioassays are still in progress. However, positive transmissions have already been observed for samples collected in ten out of the 13 ewes, including four ewes with healthy mammary glands (absence of lesions at macroscopic and microscopic examination and normal somatic cell counts [23],[24]).

**Table 3:** End-point titration of a brain homogenate (posterior brainstem- 12.5% weight/volume homogenate) in Tg338 mice.

Dilution	Number of positive mice	Incubation period in days (mean+/−SD)*
neat	6/6	221+/−20
10^−1^	6/6	348+/−16
10^−2^	12/12	481+/−32
10^−3^	10/12	594+/−34
10^−4^	7/12	713+/−43
10^−5^	3/12	805, 824, 852*
10^−6^	0/12	>900

The donor ewe was born and bred in the Langlade Flock. This ewe was at the terminal stage of Scrapie at the moment of culling. Each mouse was intracerebrally inoculated with 20 µl of homogenate. Mice were considered positive when abnormal PrP deposition was detected in brain. Incubation periods are presented as mean+/−SD except for that dilution with which less than 20% of mice were found positive. In that case (*) incubation times of the positive mice are individually presented.

**Figure 3:**
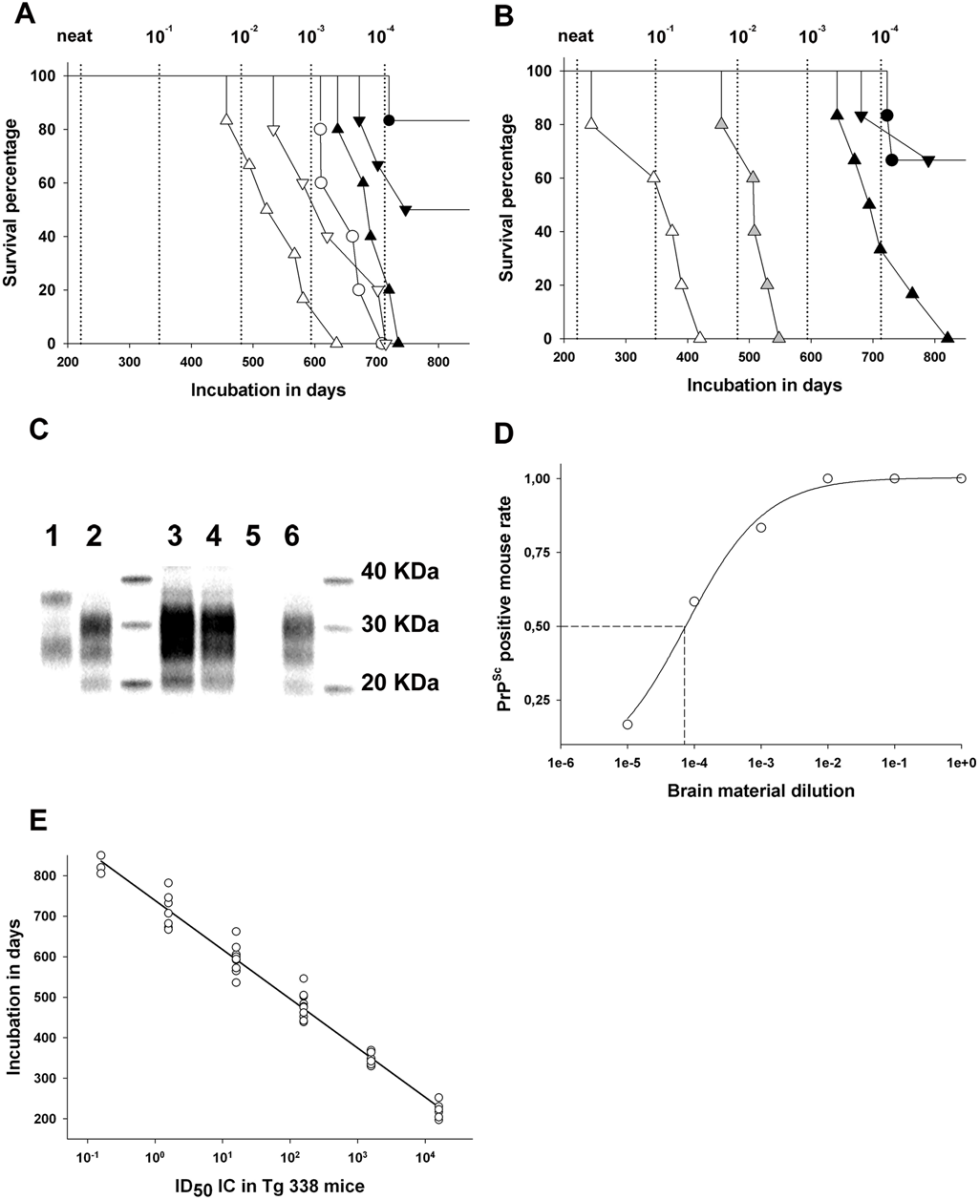
Infectivity testing in a reference brain sample and colostrum/milk fractions from scrapie incubating ewes. (A,B) Survival curve in Tg338 mice (transgenic mice over-expressing ovine VRQ PRP allele) intracerebrally inoculated with colostrum (A) and milk (B), collected from ewes incubating scrapie. Samples were first fractionated into cellular pellet (▵), cream (▿), and casein whey (○). An immunoprecipitation of PrP on magnetic beads coated with anti-PrP antibodies was then carried out. Beads from each fraction were inoculated into five or six Tg338 mice. (A) Colostrum fractions from a ewe harbouring mammary ectopic lymphoid follicles associated with Maedi lesions (white symbols) and from a ewe with a healthy mammary gland (black symbols). (B) Milk fractions from the same ewes as in A (black symbols and white symbols) and of the cellular fraction from a second scrapie incubating ewe with a healthy mammary gland (grey symbols). The experiment was terminated after 900 days (normal Tg338 mouse lifespan). Incubation periods have to be compared to those of successive 1/10 dilutions of brain (obex- vertical dotted lines) material from a sheep clinically affected with scrapie. The start point (neat) corresponds to the inoculation of 2.5 µg of brain tissue per mice. (C) Western-blotting (anti-PrP SHa31 antibody) of without (lane 1) and with (lane 2) PK treatment of brain material from a Tg338 mouse inoculated with scrapie positive brain (10^−3^ diluted); (lanes 2–6) PK digested brain material from mice inoculated with milk and colostrum cellular fraction – (lane 3) milk from a ewe with a healthy mammary gland – (lane 4) colostrum from a ewe with a healthy mammary gland – (lane 5) milk from TSE free control – (lane 6) colostrum from a Maedi affected (ectopic lymphoid follicle) ewe. (D) Intracerebral end point titration of a 12.5% obex homogenate, prepared from a terminally scrapie affected sheep (Langlade isolate), in a Tg338 mouse model. This titration allowed the determination of the infectious dose 50 (ID_50_) of the brain sample (10^6.8^ ID_50_/g), see the text. (E) Variation of the incubation period as a function of the infectious dose inoculated intracerebrally in Tg338 mice (obex – Langlade isolate), see the text.

Definitive data are now available for different fractions prepared from five animals. Two of these were TSE-free control sheep and three were scrapie incubating ewes - in one of which numerous PrP^Sc^ positive ectopic lymphoid follicles were seen in the mammary parenchyma while the mammary glands appeared normal in the other two (Figure 3A and 3B and Table 4). No transmission or PrP^Sc^ accumulation was observed in mice inoculated with fractions from the two control ewes (>850 days post inoculation). Surprisingly, TSE clinical signs and PrP^Sc^ accumulation were observed in mice inoculated with colostrum and milk fractions prepared from all three scrapie incubating ewes (Figure 3A, 3B, and 3C), indicating that prion infectivity had accumulated in colostrum and milk, even in the absence of detectable lesions in mammary glands.

**Table 4:** Estimation of infectious titre in colostrum and milk from scrapie incubating ewes with apparently healthy mammary glands or lymphoproliferative mastitis (consecutive to Maedi infection).

Ewe	Ecto. Lymph. follicles	Fraction		Quantity of material submitted to IP	Starting whole milk volume	Pos mice	Incubation period in days (mean+/−SD)	Estimated infectious titre (ID_50_ IC in Tg 338)	Global infectious titre/ml
**0942**	**pos**	**colostrum**	Cell pellet	10^7^ cells	10 ml	6/6	524+/−45	85	10^1.2^ /ml
			Casein whey	3.6 ml	3.6 ml	6/6	609+/−81	15	
			Cream	1.3 ml	20 ml	6/6	612+/−62	15	
		**Milk**	Cell pellet	1.3 10^6^ cells	30 ml	5/5	355+/−58	1250	10^1.6^ /ml*
			Casein whey	3.6 ml	3.6 ml	N.A	N.A (>400)		
			Cream	1.3 ml	38 ml	N.A	N.A (>400)		
**0248**	**neg**	**colostrum**	Cell pellet	6 10^6^ cells	8 ml	6/6	685+/−39	5	10^0.3^ /ml
			Casein whey	3.6 ml	3.6 ml	3/6	-	<1	
			cream	1.3 ml	17 ml	1/6	-	<1	
		**Milk**	Cell pellet	10^6^ cells	10 ml	6/6	717+/−45	2	10^0.1^ /ml
			Casein whey	3.6 ml	3.6 ml	3/6	-	<1	
			cream	1.3 ml	35 ml	1/6	-	<1	
**0370**	**neg**	**Milk**	Cell pellet	510^5^ cells	7 ml	6/6	509+/−34	90	10^1.1^ /ml*
			Casein whey	N.A	-		N.A		
			cream	N.A	-		N.A		

For each fraction (cell pellet, casein whey, cream) the quantity of the material submitted to immunoprecipitation process is detailed and linked to the initial volume of colostrum or milk from which it was prepared. In samples for which a 100% attack rate was observed, mean incubation period were used to estimate the infectious titre (Figure 3E). For each considered fraction the infectious titre per ml of starting material was calculated. The global infectious titre per ml of colostrum and milk was finally obtained by adding the value corresponding to each fraction.

N.A: not available at the moment of writing. *Infectivity was estimated from the only those fractions for which results are available. Consequently the calculated infectious titre/ml of milk is certainly underestimated.

All tested colostrum and milk fractions (cell pellet, casein whey and cream) transmitted disease. However, in samples for which all three fractions results are available, the cellular pellet transmitted disease with a higher attack rate and/or shorter incubation period than casein whey or cream (Figure 3A and 3B and Table 4). Moreover, incubation periods, recorded for colostrum and milk from ewes with ectopic mammary lymphoid follicles, were shorter than those of ewes displaying healthy mammary glands (Figure 3A and 3B and Table 4). This last observation could suggest higher infectivity shedding in cases of chronic lympho-proliferative mastitis.

In order to estimate the infectivity load of the colostrum and milk fractions, a brain homogenate (obex) from a terminally scrapie affected Langlade VRQ/VRQ ewe was end point titrated in the Tg338 mouse model (IC route) (Table 3). According to these data the infectious dose 50 (ID_50_) of the 12.5% (weight/volume) brain homogenate was estimated to be 10^4.2^ ID_50_ per 20 µl, ie 10^6.8^ ID_50_ per gram (Figure 3D). Using these titration data, a function correlating the observed incubation period in mice inoculated with the infectious dose was computed (Figure 3E). This function was then used to estimate the infectious content of colostrum and milk samples on the basis of the observed incubation period in Tg338 mice (Table 4). Using this approach, and keeping in mind the limited numbers of samples for which definitive results are currently available, the infectious titre in colostrum and milk samples was estimated to range between 10^0,1^ and 10^1,6^ ID_50_/ml (IC route in Tg338), which would be comparable to the infectious load contained respectively in 0.2 µg and 6 µg of the positive reference brain material (10^6.8^ ID_50_/g IC route in Tg338).

## Discussion

Failure to transmit TSE using milk from scrapie affected or incubating ewes in conventional rodents models, has been previously reported [7]. In our study, the use of a transgenic mouse model expressing the ovine PRP gene reduced or abolished the species barrier phenomenon [25] and could explain, at least partially, the positive results we obtained. However the low infectivity level we measured (10^0.1^ to 10^1.6^ ID_50_/ml) indicates that, even in this transgenic animal model, direct inoculation with 20 µl per mouse of whole milk or colostrum would be unlikely to transmit disease. It also means that, despite no PK resistant PrP being detected in milk and colostrum fractions, the approach we used (i.e immunoprecipitation of all detectable PrP on beads) was sufficiently efficient to concentrate infectivity. Measurements of the concentrative efficacy of this method are currently under investigation and will be reported elsewhere.

A recently published study reported the successful transmission of scrapie to lambs through consumption of colostrum/milk collected from ewes at the late incubation or clinical stage of the disease [11]. This study was the first to identify Prion presence in mammary secretions from Scrapie affected ewes. However, because of its design, this study did not elicit information as to which fraction, colostrum or milk, induced disease transmission. Moreover, since lateral contamination occurred between lambs and in some cases several ewes were used to feed a lamb, it was not possible in this experiment to clearly determine which of the donor ewes were shedding infectivity in colostrum/milk. The data we provided here brings definitive and unambiguous evidence of the presence infectivity in both milk and colostrum from naturally incubating scrapie ewes. In our model, infectivity was detected up to 20 months before clinical disease onset and a majority of ewes (10 out of 13 at the moment of writing) were demonstrated to have shed prion infectivity in their milk.

Taken together, the results reported by Konold et al. [11] and those obtained in our study, raise the issue of the use of sheep milk or milk by-products for animal feeding. Currently ruminants' milk represents a major source of protein in milk-replacer and feedstuffs used in a variety of farm animal species. The use of TSE incubating ewe milk in such products could give rise to dietary exposure of animals both intra and interspecies. Given the low level of infectivity apparent in milk and the species barrier phenomenon, the interspecies transmission risk associated with ewe milk certainly remains limited. However, in the current stage of knowledge, the possibility of such transmission cannot be ruled out.

As all scrapie samples in our study were collected from a single flock, it is likely that investigated ewes were exposed to only a limited range of TSE agents and possibly to a single one. Consequently, caution should be taken before inferring those observations to other situations. Interactions between host genotype and TSE agent are known to impact on the kinetics of prion dissemination in ewes. Such interactions could also influence, not only how early the shedding of infectivity via colostrum and milk takes place but also the levels of infectivity.

Prion infectivity was detected here in sheep with healthy mammary glands as well as those with chronic lymphoproliferative mastitis. However, our preliminary results suggest that the presence of ectopic lymphoid follicles in ewes with lymphoproliferative mastitis could increase prion shedding in milk. Acute and subacute bacterial mastitis are extremely common in dairy animals and can impact on milk composition [26]. The effect of these conditions on milk prion shedding was not addressed in this study and remains to be evaluated.

Our study was carried out in a flock affected by Maedi-Visna Lentivirus. This non oncogenic retrovirus is largely spread in sheep population even if its exact prevalence remains difficult to evaluate. In several countries or sheep production areas, up to 70% of flocks were reported to be infected [27]. While Maedi-Visna Lentivirus generally induces a persistent infectious with no associated pathology, it can cause, in a fraction of infected individuals, lymphoproliferative changes including a diffuse interstitial infiltration and/or peri-ductal follicle-like aggregations in the mammary gland. In such affected ewes disease has a subclinical course with secretion of apparently normal milk [28],[29].

A potential enhancement of the prion infectivity shedding in milk from persistently Maedi Virus infected ewes (with no pathological manifestations) cannot be ruled out [30]. However, because of the relative high prevalence of such infection in dairy ewes production areas, such hypothetical effect would not impact, in our opinion, on the significance of our observations.

There are major differences in terms of peripheral pathogenesis between BSE in cattle and TSE in other ruminants. In cattle BSE, peripheral tissues PrP^Sc^ accumulation and infectivity is marginal and this is particularly true of lymphoid tissues [31]. Such differences prevent the observations reported here in sheep being directly extrapolated to BSE in cattle. Nevertheless, these results clearly call for the re-examination of milk from BSE affected cattle for the presence of prions.

Finally, the consequences for humans of the presence of prions in sheep milk should certainly be given consideration. However, it is our opinion that its relative impact on global TSE dietary exposure is of lower magnitude than other prion sources, such as lymphoid tissues from small ruminants incubating TSE [32],[33].

## Methods

### Scrapie affected animals and Maedi PCR diagnosis

Scrapie positive ewes included in this experiment were all Romanov sheep born and bred in the Langlade flock. In this flock a natural scrapie epidemic has been occurring at a high incidence since 1993 [12].

Since 1997, all animals belonging to this flock are:

- genotyped at two months of age for codon 136 (A/V),154 (R/H) and 171 (Q/H/R) of the PRP gene by the SNP taqman probe method (Labogena, Jouy en Josas).
- necropsied with collection of central nervous system, lymphoid tissues and several other tissues (including mammary gland). Samples are both formalin fixed/paraffin embedded and frozen stored. The retrospective study involved a set of samples collected between 2003 and 2006. Susceptible genotype sheep included in this retrospective study were, in the majority, clinically suspect for scrapie at the time of culling.

For the prospective study a group (n = 13) of Langlade ewes, having susceptible genotypes ARQ/VRQ and VRQ/VRQ, was constituted. In the first 12 hours post lambing, 5 to 20 ml of colostrum was collected in TSE free conditions, the lambs having been separated for 4 hours from the ewes. Similarly, at 20 days post lambing, individual samples of milk (10 to 50 ml) were collected.

Milk from two VRQ/VRQ cheviot TSE free sheep (Arthur Rickwood, UK) was collected and included in the study as a negative control. This flock is the only source in Europe of sheep free of classical scrapie. The TSE-free status of the dams was confirmed by post-mortem laboratory examination.

PCR detection of Maedi virus was carried out on DNA extracted from mammary tissue. Primers (Forward:CCACGTTGGGCGCCAGCTGCGAGA-Reverse:TGACACAGCAAATGTAACCGCAAG) and PCR conditions (40 cycles – annealing 58°C) were those published by Sonigo et al [34]. Reference positive case and a negative controls were included in each PCR run. PCR products (291 bp) were migrated on a 2% agarose TBE gel. Positive samples were identified on the basis of PCR product size (by comparison with a positive control plasmid).

### PrP^Sc^ distribution in ARQ/VRQ and VRQ/VRQ sheep organism

An ARQ/VRQ (n = 72 – age 18 months) ewe cohort was inseminated with semen from a single VRQ/VRQ ram. Natural mating or contact between ewes and rams was strictly avoided. The resulting Lambs (birth cohort September 2003) were PRP genotyped at the age of 2 months. Groups of 4 VRQ/VRQ and 4 ARQ/VRQ animals were euthanazied by exsanguination after intravenous pentobarbital (DOLETHAL^ND^, 10 mg/kg) injection at 4 months, 7 months, 10 months, 13 months and 20 months of age. Scrapie clinical signs occurred in homozygote VRQ animals at 20 months of age. A final group of 4 ARQ/VRQ animals was euthanized at 32 months, when first clinical signs where observed. At necropsy, lymphoid, digestive tract and central nervous system (CNS) tissues were sampled extensively from each animal; these were formalin fixed and processed for PrP^Sc^ IHC detection.

### Tissue processing and immunohistochemistry (IHC) detection

This method was performed as previously described [16]. PrP^Sc^ IHC detection was first performed using 8G8 antibody raised against human recombinant PrP protein and specifically recognising the 95–108 amino acid sequence (SQWNKP) of the PrP protein.

For each sample a negative serum control was included, in which the primary antibody was either omitted or replaced by purified mouse Ig2a serum. In addition, anti-PrP monoclonal antibodies were replaced by isotype-matched monoclonal antibodies irrelevant to the protein under investigation. PrP^Sc^/CD68 double labelling was performed as previously described [16], using KiM6 monoclonal mouse anti human CD68 (Serotec, London, UK) and a rabbit anti-PrP serum (R521- diluted 1/1000– CIV, the Netherlands). For double-labelling, cross-reactivity controls were performed, in order to verify the absence of inter-species reactivity of secondary antibodies toward primary antibodies. The absence of affinity between the two secondary antibodies was also checked.

### Paraffin embedded tissue blot (PET blot)

PET blot was performed using a method previously described [35]. Immunodetection was carried out using SHa31 monoclonal antibody (4 µg/ml), which recognizes the 145–152 sequence of PrP (YEDRYYRE), followed by application of an alkaline phosphatase labelled secondary antibody (Dako ref D0314 – 1/500 diluted). Enzymatic activity was revealed using NBT/BCIP substrate chromogen. For each tissue sample, serial sections of 4 µm thickness for PET blot and 2 µm for IHC were collected onto membranes or glass slides respectively. Both methods were then carried out and the resulting preparations were subject to comparison. This experimental design allowed the use of shape and localization of labeling on the IHC sections to identify the nature of PET blot PrP^Sc^ positive structures.

### Milk and colostrum fraction preparation

Milk and colostrum samples were all collected under TSE sterile conditions. An aliquot of each collected sample was submitted to a standard somatic cell count (SCC) (by flow cytometry) by an state accredited laboratory (LIAL, Auch, France).

Each sample type was first diluted (1/2 for milk and 1/5 for colostrum) in PBS containing 10% acid-citrate-dextrose (Sigma-Aldrich ref C3821) and 10 mM EDTA-2K and homogenized by inversion. After standing for 30 min at 4°C, the cream was collected with a single use spatula. A Pasteur pipette was then passed through the layer of residual cream and the liquid was aspirated; this was passed through a 200 µm filter and collected in 50 ml tubes. The liquid sample was then centrifuged at 2000 rpm at 4°C for 5 minutes. The supernatant (casein-whey) was collected and stored frozen while cell pellets were transferred to a new 50 ml tube. The cells were washed three times by successive centrifugation/resuspension phases in PBS and counted in a Malassez cell before frozen storage.

### PrP immunoprecipitation in colostrum and milk fractions

Cells and whey were extracted for 10 min at 37°C in NP40/DOC buffer (NP40 0.5% (V/V), deoxycholate 1% (W/V), EDTA 10 mmol.L^−1^, NaCl 150 mmol.L^−1^, Tris 10 mmol.L^−1^ pH 7.4). The cream was extracted for 10 min at 37°C in sarkosyl buffer (N-lauroyl sarcosine 10% (W/V), EDTA 10 mmol.L^−1^, NaCl 150 mmol.L^−1^, Tris 20 mmol.L^−1^ pH 7.4). Three different monoclonal antibodies (SHa31, SAF-34 and βS-36) [20],[36] were immobilized by covalent coupling to magnetic beads (Dynal Biotech) and used to perform immunoprecipitation. Two successive immunoprecipitations (overnight 4°C), using a mixture of the three different antibody coated beads, were performed and any non-immunoprecipitated residual PrP in the supernatant was measured using a two site sandwich immunoassay (capture antibody 11C6 [20], tracer antibody Bar-224 [20]- CEA Saclay).

Whenever the concentration of residual PrP was in excess of 5% of that of the unimmunoprecipitated control a third immunoprecipitation (2 h/RT) was performed.

Beads were washed three times (two washings in PBS/Tween 1% and one in PBS) before re-suspension in the appropriate volume of 5% glucose. The concentration factor was 15 times for cream (equivalent to 300 µL inoculated per mouse) and 30 for whey (equivalent to 600 µL inoculated per mouse).

### Colostrum and milk bioassay

Bioassay experiments were carried out in ovine VRQ PrP transgenic mice (Tg338), which are considered to be highly efficient for the detection of sheep scrapie infectivity [22]. Immunoprecipitated cream, casein whey and cell pellet fractions were re-suspended in 130 µL of sterile 5% glucose. Six mice were intracerebrally inoculated with each sample (20 µL). Colostrum inoculations were carried out in UMR INRA ENVT 1225 (Toulouse, France) facilities while milk fractions were tested at both the NVI (Oslo, Norway) and at INRA IASP (Tours, France). Samples from each animal were inoculated on different days so as to avoid any risk of cross contamination. Mice were then clinically monitored until the occurrence of TSE clinical signs, at which time they were culled. CNS and spleen samples were individually collected and Western blot (WB) tested. Mice inoculated with control TSE free sheep tissue and milk fractions were culled 950 days post inoculation. The majority of the bioassays are still in progress.

### Estimation of infectious titre

A sample of obex from a VRQ/VRQ Langlade sheep, clinically affected with scrapie, was homogenized (12.5% weight/volume) before intracerebral inoculation (20 µl) of successive 1/10 dilutions in groups of Tg 338 mice (6 or 12 mice). The Infectious Dose 50 of this brain homogenate was determined using a four parameter logistic regression approach, excluding the last point of end titration (no positive animals).

Incubation periods in mice were then plotted on a graph, the different Infectious Dose parameters being calculated for each dilution. A linear regression function was computed using this dataset and then used to estimate the infectious titre (number of Infectious Dose 50) contained in the colostrum and milk samples.

### PrP^Sc^ Western-blot detection (WB)

A Western blot kit (TeSeE Western Blot, Bio-Rad) was used following the manufacturer's recommendations.
